# Tobacco Smoking and Gray Matter Volume in Individuals at Clinical High Risk for Psychosis: A Longitudinal Magnetic Resonance Imaging Study

**DOI:** 10.1016/j.bpsgos.2025.100539

**Published:** 2025-05-27

**Authors:** Merel Koster, Marieke van der Pluijm, Maura Fraikin, Guido van Wingen, Elsmarieke van de Giessen, Lieuwe de Haan, Jentien Vermeulen, Tim Ziermans

**Affiliations:** a Department of Psychiatry, Amsterdam University Medical Center (UMC), University of Amsterdam, Amsterdam, the Netherlands; b Department of Radiology and Nuclear Medicine, Amsterdam UMC, University of Amsterdam, Amsterdam, the Netherlands; c Department of Psychology, University of Amsterdam, Amsterdam, the Netherlands

**Keywords:** Clinical high risk, Gray matter volume, Magnetic resonance imaging, Nicotine, Psychosis, Tobacco smoking

## Abstract

**Background:**

Smoking is pervasive in young adults before psychosis onset and has been linked to worse clinical outcomes. Research suggests that smoking may play a role in psychosis pathogenesis, as increased smoking and gray matter reductions are associated with psychosis risk. However, a direct relationship in people at clinical high risk for psychosis (CHR-P) has not been established.

**Methods:**

3T structural magnetic resonance imaging scans from the NAPLS-3 (North American Prodrome Longitudinal Study 3) were used. At baseline, 432 CHR-P nonsmokers and 110 CHR-P smokers were included, totaling 1617 scans across 2-, 4-, 6-, and 8-month follow-ups. Baseline gray matter volume differences between smoking and nonsmoking CHR-P were assessed with voxel-based morphometry. Linear mixed-effects models were used to examine association between smoking and gray matter volume across age in the superior frontal gyrus, anterior cingulate cortex, and insula. CHR-P individuals were categorized by tobacco use (no, low, high) to explore dose-response associations.

**Results:**

At baseline, no significant differences in gray matter volume were observed between smoking and nonsmoking CHR-P individuals, regardless of the tobacco use level. Longitudinal analyses showed no significant group or group × age associations with gray matter volume between the 2 groups.

**Conclusions:**

We observed no cross-sectional or longitudinal associations over 8 months between smoking and gray matter volume in CHR-P individuals. This suggests that no tobacco-related associations with gray matter volume reductions are evident yet in this vulnerable group, both in terms of psychosis and addiction risk. However, low smoking frequency and intensity in the current sample warrant further research with CHR-P individuals who are heavier smokers.

## Plain Language Summary

Smoking is pervasive in individuals at clinical high risk for psychosis (CHR-P) and is linked to worse clinical outcomes. Research suggests that smoking may play a role in psychosis pathogenesis. However, a direct relationship in CHR-P individuals has not been established. Using magnetic resonance imaging scans from 432 smoking and 110 nonsmoking CHR-P individuals, we investigated gray matter volume differences between groups at baseline and over an 8-month follow-up. Results showed no significant differences in gray matter volume between groups either at baseline or over time. This suggests that mild tobacco use is not yet associated with structural brain changes in CHR-P individuals.

The prevalence of tobacco smoking is high in people at clinical high risk for psychosis (CHR-P) (1,2), and it is even higher among individuals diagnosed with psychotic disorders (3,4) and is associated with worse clinical outcomes (5). Research even suggests that smoking is associated with the pathogenesis of psychosis (6, 7, 8, 9, 10). Specifically, tobacco smoking is associated with increased risk of psychosis (6,8,9), and daily use is associated with an earlier age at onset of psychotic illness (6). Furthermore, a dose-response relationship between tobacco smoking and the incidence of schizophrenia has been reported (7,8), and one study demonstrated that there is at least in part a causal effect of smoking on schizophrenia (10). People at CHR-P are at a 20% to 30% risk of developing psychosis within the next 3 years (11), and defined as exhibiting attenuated psychotic symptoms or a decline in functioning or having a family history of psychotic disorders (12,13). The high prevalence of smoking in young individuals at CHR-P is alarming, as research suggests that continued tobacco smoking in CHR-P individuals is linked to a worsening of psychotic symptoms, while reducing the number of cigarettes smoked is associated with improvements in affective symptoms (14). However, the underlying mechanisms for the association between smoking and psychosis remain unclear.

Neuroimaging research can offer insights into the underlying neurobiological mechanisms and consequences of smoking. Structural neuroimaging studies have consistently demonstrated widespread gray matter volume reductions in schizophrenia (15,16), with similar but less pronounced effects in CHR-P individuals who develop psychosis (17,18). Structural imaging studies that have examined the association of smoking and brain structure in patients with schizophrenia have suggested widespread independent and additive reductions in gray matter in relation to smoking and schizophrenia (19). However, no studies have specifically investigated the association of smoking and brain structure in CHR-P individuals.

However, separate studies have revealed gray matter reductions in overlapping brain regions associated with both smoking and CHR-P. These regions include the superior frontal gyrus (SFG) (20, 21, 22), which is involved in high-level cognitive functions such as working memory and executive function; the insular cortex (22, 23, 24, 25), which is involved in emotional and sensorimotor processing; and the anterior cingulate cortex (ACC) (23,26,27), which is involved in emotion regulation and reward-based decision making (28). Tobacco smoking introduces high levels of oxidizing agents, which can lead to a state of surplus of reactive oxidants and relative shortage of antioxidants, also known as oxidative stress. Oxidative stress causes damage to lipids, proteins, and DNA and may eventually result in cell death (29), which is thought to be linked to brain volume decreases (30,31). Furthermore, oxidative stress can subsequently trigger neuroinflammation through a cytokine-mediated immune response. Over time, the cumulative neurotoxic effects of smoking may result in reduced brain volume (32). Adolescence and young adulthood are critical periods for brain maturation, marked by ongoing myelination and synaptic pruning. Smoking during this time may accelerate brain aging, disrupt maturation trajectories, or contribute to structural abnormalities (33). It may also interact with pre-existing brain differences to influence clinical outcomes and psychosis risk. Because the SFG, insula, and ACC are implicated in both schizophrenia and smoking, and are still undergoing structural refinement during this developmental window (34), their integrity may be particularly relevant in CHR-P individuals who smoke. Investigating the influence of smoking on brain structure in CHR-P individuals could enhance our understanding of the role of smoking in early psychosis, further supporting targeted interventions before the onset of psychosis. In addition, longitudinal studies are essential to be able to differentiate between pre-existing anatomical and physiological differences and the (long-term) neurotoxic consequences of smoking and thus inform needed preventive strategies.

Therefore, we aimed to investigate the association of smoking and brain volume over time in CHR-P individuals from the multisite dataset of NAPLS-3 (North American Prodrome Longitudinal Study 3) (35). First, we assessed cross-sectional whole-brain differences between CHR-P smokers and CHR-P nonsmokers. Second, we examined longitudinal associations between smoking and gray matter volume changes in the SFG, ACC, and insula over an 8-month period. Furthermore, we explored cortical thickness and surface information (i.e., gyrification index) to complement our volume analysis for a more comprehensive understanding of the association between smoking and structural changes in CHR-P individuals. Based on the previous findings of overlapping brain regions in both smokers and CHR-P individuals, we expected reductions in in the SFG, ACC, and insula in CHR-P smokers compared with CHR-P nonsmokers.

## Methods and Materials

### Participants

This study used data collected in NAPLS-3, aimed at investigating early detection and prediction of psychosis onset (35). Conducted from 2015 to 2020, the study incorporated data from 9 different sites across the United States. A detailed overview of study procedures is provided in Addington *et al.* (35).

Participants included CHR-P individuals ages 12 to 30 years at baseline. Participants were seeking help and either self-referred or referred through health care providers, educators, or social service agencies and met criteria for psychosis-risk syndrome as assessed by the Structured Interview for Psychosis-risk Syndromes (36). Attenuated psychotic symptoms were rated on the Scale of Psychosis-Risk Symptoms (36). Nineteen items distributed across 4 domains (positive, negative, disorganization, and general symptoms) were scored as follows: 0 = absent, 1 = questionably present, 2 = mild, 3 = moderate, 4 = moderately severe, 5 = severe but not psychotic, and 6 = severe and psychotic. Exclusion criteria comprised 1) current or lifetime DSM-5 Axis I psychotic disorder diagnosis, including affective psychoses; 2) IQ < 70; 3) history of a central nervous system disorder; or 4) psychosis-risk symptoms attributable to an Axis I disorder. Institutional review boards at all sites approved the study procedures, and informed consent was obtained from all participants or from their parents if participants were <16 years of age. In this study, we only included participants with available measurements on smoking behavior and a baseline magnetic resonance imaging (MRI) assessment.

### Study Design and Measures

Demographic characteristics were registered at baseline, while neuroimaging and clinical assessments occurred at baseline and 2, 4, 6, and 8 months follow-up. Smoking behavior and alcohol and cannabis use were evaluated using the Alcohol and Drug Use Scale (37). Tobacco smoking frequency was rated as follows: 0 = no use, 1 = occasionally, 2 = <10 cigarettes per day, 3 = 10 to 25 cigarettes, 4 = >25 cigarettes per day. Participants were considered smokers if they reported smoking at least occasionally (score ≥ 1). Cannabis and alcohol use were rated as follows: 0 = no use, 1 = once or twice per month, 2 = 3 to 4 times per month, 3 = 1 to 2 times per week, 4 = 3 to 4 times per week, 5 = almost daily in the past month. Participants were classified as cannabis or alcohol users if they reported using either substance at least once or twice per month (score ≥ 1).

### MRI Acquisition and Preprocessing

High-resolution whole-brain T1-weighted scans were acquired using 3T MRI scanners using a magnetization-prepared rapid acquisition gradient-echo (MPRAGE) sequence and a standardized set of imaging parameters across all sites (38). Acquired structural MRI data were preprocessed using the Computational Anatomy Toolbox (CAT12; version r2170) (39) for SPM12 (Wellcome Department of Cognitive Neurology) running in MATLAB (version R2022a; The MathWorks, Inc.). For the baseline cross-sectional analysis, we utilized the default preprocessing stream provided by CAT12. After initial bias correction and noise reduction, images were segmented into gray matter, white matter, and cerebrospinal fluid using CAT12’s unified segmentation approach. Extracted gray matter maps were normalized to a 1.5-mm Montreal Neurological Institute template using Geodesic shooting registration, which uses affine registration followed by nonlinear registration. The segmented images were modulated using the Jacobian determinants to adjust for volume changes due to normalization. Finally, the normalized and modulated images were smoothed with a 7-mm full width at half maximum (FWHM) Gaussian kernel. An absolute threshold mask of 0.1 was applied to the gray matter images. After preprocessing, quality was checked by visual inspection and using the quality measures offered by CAT12 (i.e., gray matter homogeneity check).

For longitudinal data, images were segmented according to the longitudinal processing pipeline implemented in CAT12 with default parameters. Lastly, we extracted mean volumes (in mL) within bilateral regions of interest (ROIs) defined by the Automated Anatomical Labeling atlas using CAT12 (40). Our initial baseline analysis revealed no significant differences, which prevented us from identifying data-driven ROIs for longitudinal analyses. As per our preregistered plan, in the absence of such findings, we selected ROIs with overlapping gray matter abnormalities in both smokers and CHR-P individuals, including the prefrontal cortex and the insula. Within the prefrontal cortex, we focused on the SFG and also included the ACC. Thus, our final ROIs were the SFG, insula, and ACC.

### Cross-Sectional Voxel-Based Morphometry

We preregistered our analysis plan and hypotheses prior to conducting any analyses (https://aspredicted.org/bvn2-2dmk.pdf). For deviations from the preregistered analysis plan, please see Supplemental Section S1. A priori, age (41), sex (42,43), and total intracranial volume (TIV) (44) were selected as covariates for all analyses due to their potential influence on gray matter volume. In addition, given the established association between tobacco smoking and cannabis use (45), we also included cannabis status (user vs. no user) as a covariate. Lastly, the MRI acquisition site was included as covariate to account for potential intersite variabilities (dummy coded). A whole-brain voxel-based morphometry was conducted using CAT12 to explore regional differences in gray matter volume between CHR-P smokers and CHR-P nonsmokers at baseline. An analysis of covariance (ANCOVA) was utilized, incorporating the covariates age, age^2^, sex, MRI site, TIV, and cannabis use (yes/no). Significance was estimated using the threshold-free cluster enhancement (TFCE) statistic with 5000 permutations, avoiding an arbitrary threshold for initial cluster formation (46). Results were considered significant at *p* < .01, familywise error (FWE) corrected. If a statistically significant cluster was found, the binarized clusters were saved as a mask to serve as a basis for subsequent longitudinal ROI analyses.

To address the effects of an unbalanced distribution in CHR-P smokers and nonsmokers, we conducted a sensitivity analysis in equally distributed samples matched for age, sex, and cannabis use. These analyses were not part of the preregistered plan but were added to enhance the robustness of our findings. Matching was performed with the MatchIt package (47) using R (version 4.3.2; R Core Team, 2022) in RStudio (version 2023.12.1; RStudio Inc.).

### Exploratory Analyses

Exploratorily, whole-brain surface-based morphometry was conducted using CAT12 to analyze differences in cortical thickness and surface information (obtained in the form of gyrification index) between CHR-P smokers and nonsmokers at baseline. Resampled surface data were smoothed using a 12-mm and 20-mm FWHM Gaussian kernel for cortical thickness and gyrification index, respectively. An ANCOVA was utilized that incorporated the covariates age, age^2^, sex, MRI site, and cannabis use (yes/no). Significance was estimated using the TFCE statistic with 5000 permutations. Results were considered significant at *p* < .01 (FWE corrected). Anatomical regions emerging from the between-group comparisons were labeled using the Desikan-Killiany atlas (DK40) as implemented in CAT12 (48).

Exploratory analyses of baseline gray matter volume, cortical thickness, and gyrification index differences between CHR-P and control participants were carried out in accordance with preregistration but presented in the Supplement as they do not directly address smoking-related questions (see the Supplement for the results and discussion).

### Statistical Analysis

Statistical analyses were performed using RStudio, and differences were considered statistically significant at *p* < .05. For longitudinal analyses, linear mixed-effects (LME) models were used to assess associations between smoking and gray matter volume trajectories across age in selected ROIs. Age, instead of the preregistered variable time point, was included as a predictor to account for the substantial brain developmental changes that occur during adolescence and young adulthood, which characterize our sample. Data were included in the model if both MRI and smoking status data were available for at least 1 time point, as mixed modeling enables calculating valid estimates of missing data under the assumption of missing at random. Participants were classified as smokers from the moment they started smoking, and this classification remained in place for all subsequent time points, as smoking may induce lasting changes (49) that are unlikely to reverse within a short follow-up period, such as a temporary 2-month cessation. LME models were implemented in RStudio using the lmer function from the lme4 package (50). We added random intercepts for participants and random slopes for age at the participant level in all models. Smoking status and its interaction with age and the covariates sex, TIV, cannabis status, and MRI site were added as fixed effects. Continuous variables (i.e., age and TIV) were centered to improve model performance and interpretability (51). A step-by-step procedure was used to incorporate each variable, while model fit was evaluated using the Akaike information criterion for comparison. Final models were fitted using restricted maximum likelihood estimation. *p* Values were calculated according to the Kenward-Roger method, which has been shown to have low type I error (52). Residual plots were visually inspected to evaluate deviations from homoscedasticity or normality. In case of violation of assumptions, data were transformed. When transformations did not improve violated assumptions, we proceeded with the untransformed data, as LME models have been shown to be relatively robust against violation of the normality assumption (53). To correct for multiple testing, false discovery rate (FDR) correction (Benjamini-Hochberg) was used for the 3 ROIs (SFG, ACC, and insula) in all main analyses. Cohen’s *f*^2^ was calculated as effect size for ROI analyses, with *f*^2^ ≥ 0.02, *f*^2^ ≥ 0.15, and *f*^2^ ≥ 0.35 representing small, medium, and large effect sizes, respectively. Additionally, although not part of the preregistered plan, Bayes factors were calculated comparing the models with smoking status/frequency to the models without smoking status/frequency. Therefore, we assessed the strength of the reported evidence, ranging from a Bayes factor of 1, indicating no evidence for the null hypothesis, to a Bayes factor of <1/100, indicating extremely strong evidence for the null hypothesis (Supplemental, section S2).

First, we performed a group × age analysis among CHR-P smokers and nonsmokers to examine the association between smoking and gray matter volume across age in the SFG, ACC, and insula. Next, we performed additional LME models, substituting smoking status with past-month tobacco frequency. These analyses were not part of the preregistered plan but were added to explore possible dose-response relationships within the CHR-P group. Because of the limited statistical power due to small groups, we combined smoking frequencies 2, 3, and 4 for subsequent analysis. Therefore, groups consisted of CHR-P nonsmokers, light smokers, and moderate-to-heavy smokers.

Missing data on cannabis and alcohol use were imputed with multiple imputation by chained equations using the mice package in RStudio (54). Five datasets with a maximum of 50 iterations for each imputed dataset and the predictive mean matching method were used for imputation. Additionally, the seed value for random number generation was set to 500.

## Results

### Baseline Demographic and Clinical Characteristics

**Figure 1:**
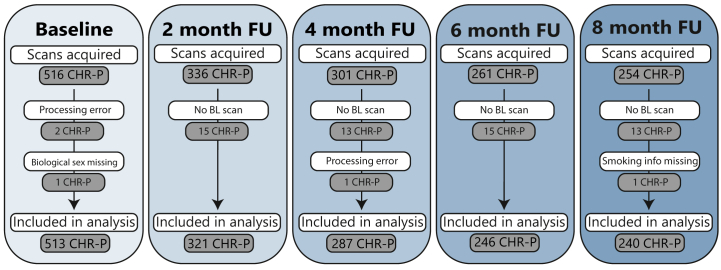
Number of participants and reasons for exclusion from the analysis. Scans of participants without baseline magnetic resonance imaging data were excluded (*n* = 15 at month [M] 2, *n* = 13 at M4, *n* = 15 at M6, *n* = 13 at M8). Moreover, we excluded participants with no information on smoking behavior (*n* = 1 clinical high risk for psychosis [CHR-P] at M8) and no information on sex assigned at birth (*n* = 1 CHR-P at baseline). Additionally, 3 participants were excluded due to preprocessing errors (*n* = 2 CHR-P at baseline, *n* = 1 CHR-P at M4). FU, follow-up.

In baseline analysis, 542 CHR-P individuals were included, comprising 110 smoking and 432 nonsmoking individuals. See Figure 1 for an exclusion flowchart and reasons for exclusion. Missing data on cannabis use were imputed for 1 nonsmoking participant at 2 time points (at baseline and month 2; data at months 4, 6, and 8 were available).

Smoking participants were significantly older, reported more years of education, used cannabis more frequently and heavily, consumed alcohol more often and in greater quantities, and were more often male than nonsmoking participants (all *p*s < .001). Smoking participants also exhibited more positive symptoms than nonsmoking participants (*p* = .012). Fifty-seven participants (11%) converted to psychosis. Baseline demographic and clinical characteristics are summarized in Table 1. In total, there were 1075 follow-up scans (Table S1). At baseline, 20% of participants smoked, which slightly increased over time to 27% at the 8-month follow-up (Table S2). In total, 27 (6%) participants started smoking, and 18 (16%) stopped sometime during follow-up.

**Table 1:** Demographic and Clinical Characteristics of CHR-P Participants at Baseline

	CHR-P, *n* = 542		*p*
	Nonsmokers, *n* = 432	Smokers, *n* = 110	
Age, Years	18.0 ± 3.90	20.4 ± 3.57	<.001^a^^,^^b^
Sex, Female	204 (47%)	31 (28%)	<.001^a^^,^^c^
Years of Education	11.3 ± 3.12	12.9 ± 2.30^d^	<.001^a^^,^^b^
Smoking Frequency			
No use	432 (100%)	0 (0%)	<.001^a^^,^^c^
Occasionally	0 (0%)	64 (58%)	
<10 cigarettes per day	0 (0%)	26 (24%)	
10–25 cigarettes per day	0 (0%)	18 (16%)	
>25 cigarettes per day	0 (0%)	2 (2%)	
Cannabis Users	73 (17%)	70 (64%)	<.001^a^^,^^c^
Cannabis Use Severity			
No use	359 (83%)	40 (36%)	<.001^a^^,^^c^
Once/twice per month	37 (9%)	25 (23%)	
3–4 times per month	8 (2%)	10 (9%)	
1–2 times per week	11 (3%)	15 (14%)	
3–4 times per week	11 (3%)	11 (10%)	
Almost daily	6 (1%)	9 (8%)	
Alcohol Users	126 (29%)	82 (75%)	<.001^a^^,^^c^
Alcohol Use Severity			
No use	306 (71%)	28 (25%)	<.001^a^^,^^c^
Once/twice per month	60 (14%)	25 (23%)	
3–4 times per month	27 (6%)	22 (20%)	
1–2 times per week	25 (6%)	15 (14%)	
3–4 times per week	10 (2%)	17 (15%)	
Almost daily	4 (1%)	3 (3%)	
Symptom Severity			
Total negative symptoms	12.1 ± 6.57^e^	12.4 ± 5.48^f^	.713^b^
Total positive symptoms	12.7 ± 3.40	13.5 ± 3.17^d^	.012^a^^,^^b^
Total general symptoms	9.25 ± 4.41^g^	9.64 ± 3.35^f^	.328^b^
Total disorganization symptoms	5.05 ± 3.26^g^	5.51 ± 3.00^h^	.164^b^
Antipsychotic Users	87 (20%)	26 (24%)	.500^c^
Antipsychotic Medication Dosage, mg/Day CPZ	146 ± 139	208 ± 257	.194^b^
Conversion to Psychosis	46 (11%)	11 (10%)	.981^c^

CHR-P, clinical high risk for psychosis; CPZ, chlorpromazine.

a Significant *p* values.

b Independent *t* test.

c χ^2^ test.

d Data were missing for 1 person.

e Data were missing for 7 people.

f Data were missing for 3 people.

g Data were missing for 8 people.

h Data were missing for 2 people.

### Cross-Sectional VBM

No significant group differences were observed between the CHR-P smokers and nonsmokers. Sensitivity analyses in equally distributed groups for CHR-P smoking and nonsmoking (*n* = 110 per group) matched for age, sex, and cannabis use similarly revealed no significant differences.

### Longitudinal VBM

**Figure 2:**
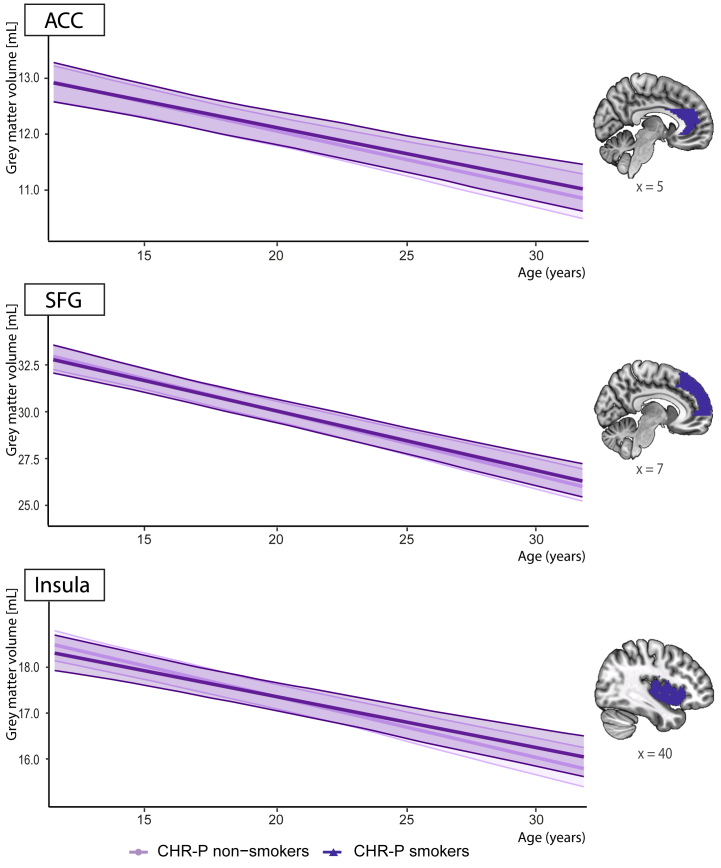
Trajectories of anterior cingulate cortex (ACC), superior frontal gyrus (SFG), and insula gray matter volumes across age per group and visualization of the regions of interest. Lines represent predicted gray matter volume values (in mL) from linear mixed-effects models including fixed effects for smoking status × age, sex, total intracranial volume, cannabis use, and magnetic resonance imaging site, with random intercepts for participant and random slopes for age. Shaded areas represent 95% CIs. Age was originally centered in months; here, actual age in years is shown for interpretability. ROIs, as defined by the Automated Anatomical Labeling atlas, are overlaid on a standardized T1-weighted image. CHR-P, clinical high risk for psychosis.

Gray matter volumes of the ACC, SFG, and insula over time are summarized in Figure 2. LME analyses showed no significant group or group × age associations in these regions between CHR-P smokers and nonsmokers (Table 2 and Tables S3–S8). However, age was negatively associated with SFG (*p*_FDR_ < .001, Cohen’s *f*^2^ = 0.81), ACC (*p*_FDR_ < .001, Cohen’s *f*^2^ = 0.33), and insula (*p*_FDR_ < .001, Cohen’s *f*^2^ = 0.50) volumes, indicating a decline over time in the CHR-P group regardless of the smoking status. Similarly, LME analyses based on smoking frequency showed no significant group or group × age associations with SFG or ACC volume, but negative associations of age with SFG (*p*_FDR_ < 0.001, Cohen’s *f*^2^ = 0.62), ACC (*p*_FDR_ < .001, Cohen’s *f*^2^ = 0.25), and insula (*p*_FDR_ < .001, Cohen’s *f*^2^ = 0.35) volumes were observed. A moderate-to-heavy smoking frequency × age association with insula volume was demonstrated but did not survive multiple comparison correction (*p*_FDR_ = .108) (Table S8). All Bayes factors were <1/100, providing extremely strong evidence for the null hypothesis that there is no association between both smoking status or smoking frequency and SFG, ACC, or insula gray matter volume.

**Table 2:** Longitudinal Analysis of Smoking and Age-Related Differences in SFG, ACC, and Insula Gray Matter Volume in CHR-P Individuals

Effects	Estimate	SE	*t*	*p*	*p*_FDR_	Cohen’s *f*^2^
SFG						
Group	0.003	0.099	*t*_1352_ = 0.029	.977	.977	0.000
Age	−0.029	0.002	*t*_246_ = −13.464	<.001^a^	<.001^a^	0.810
Group × age	0.002	0.002	*t*_970_ = 0.870	.384	.449	0.000
ACC						
Group	0.055	0.044	*t*_1322_ = 1.24	.215	.645	0.001
Age	−0.009	0.001	*t*_235_ = −8.623	<.001^a^	<.001^a^	0.330
Group × age	0.001	0.001	*t*_937_ = 0.758	.449	.449	0.000
Insula						
Group	−0.013	0.054	*t*_1392_ = −0.236	.814	.977	0.000
Age	−0.011	0.001	*t*_255_ = −10.041	<.001^a^	<.001^a^	0.500
Group × age	0.002	0.001	*t*_940_ = 1.504	.133	.399	0.003

Fixed effects in the models were smoking status × age, sex, total intracranial volume, cannabis use, and magnetic resonance imaging site. Random effects were intercepts for participants and random slopes for age.

ACC, anterior cingulate cortex; CHR-P, clinical high risk for psychosis; FDR, false discovery rate; SFG, superior frontal gyrus.

a Significant *p* values.

### Exploratory Cross-Sectional Surface-Based Morphometry

No significant differences in cortical thickness or gyrification index were found between smoking and nonsmoking CHR-P participants at baseline.

## Discussion

Because of the high prevalence of smoking in CHR-P individuals and association with worse clinical outcomes, in the current study, we sought to investigate the association of smoking and brain volume over time in CHR-P individuals. Smoking was not linked to differences in whole-brain gray matter volume cross-sectionally in CHR-P participants. Additionally, we found no significant longitudinal differences in gray matter volume within the SFG, ACC, or insula between smokers and nonsmokers or among nonsmokers, light smokers, and moderate-to-heavy smokers.

Based on the current results, it appears that infrequent or mild smoking behavior is not associated with structural differences before the onset of psychosis. This is also supported by our Bayesian analyses, which provide very strong evidence that smoking is not associated with gray matter volume. Because we observed no substantial structural reductions in gray matter volume associated with low-intensity smoking, this may point to an optimal window for offering smoking cessation support before the onset of psychosis to improve clinical outcomes and prevent structural reductions associated with smoking in patients with psychosis. The large sample size supports the robustness and reliability of our findings. This suggests that any short-term effects of smoking on gray matter volume in CHR-P individuals or pre-existing differences are likely small.

However, several important limitations that might have reduced our ability to detect smoking-related structural brain differences in CHR-P individuals should be considered. Specifically, the relatively low smoking frequency in our sample; the expected structural reductions that have commonly been observed in CHR-P populations compared with control populations (18,21,55); and baseline differences between smoking and nonsmoking participants in age, sex, and cannabis and alcohol use might have collectively contributed to the absence of significant findings. First, the smoking frequency and duration in our CHR-P sample may be too low to be associated with structural changes. Smokers were defined as individuals who smoked at least occasionally due to the small number of frequent smokers, while most studies have focused on heavier smokers (22,27). Furthermore, Van Haren *et al.* (56) found no correlation between gray matter volume and cigarette use in schizophrenia when including patients with lower smoking frequencies but observed a negative association in heavier smokers. Moreover, our sample was relatively young (mean age 18.4 years), whereas previous studies typically included older participants with longer exposure (57). The younger age could explain the lower smoking prevalence in our CHR-P group, which was unexpectedly about 20% lower than prevalence rates reported in other studies (57). Regional factors may also play a role, as smoking rates tend to be lower in the United States than in Europe and Asia (58). These combined factors might have resulted in the underlying processes not being sustained for long enough or severely enough to produce observable structural changes in the current study. Second, baseline differences in age, sex, cannabis use, and alcohol use between smoking and nonsmoking CHR-P participants must be considered. While sensitivity analyses with matched groups for these variables yielded similar results, these analyses inherently have reduced statistical power. Bayesian analyses supported the null hypothesis that smoking status and frequency are not associated with gray matter volume. However, sample composition and brain maturation effects might have masked subtle smoking-related changes. In summary, our findings suggest that smoking is not strongly linked to brain volume differences in CHR-P, but future research should include a more balanced sample with higher smoking intensity and using follow-up periods longer than 8 months to draw more definitive conclusions.

Subtle short-term differences in relatively mild smokers may be detected by other imaging techniques such as functional MRI (fMRI) or diffusion MRI. Exploring alternative neuropathways beyond structural changes is crucial for a thorough understanding of the impact of smoking in CHR-P and may further support therapeutic interventions. fMRI studies that have examined the association between smoking and schizophrenia have suggested distinct effects of smoking on neural connectivity in patients with schizophrenia (19). Furthermore, smoking among young adults is associated with increased white matter integrity (59). These findings emphasize the importance of future research utilizing these measures in studies that investigate the effects of smoking in CHR-P individuals. Furthermore, the association between smoking and (risk of) psychosis could be attributed to underlying differences in the nicotinic acetylcholine receptors (nAChRs) between patients with psychosis and control participants. nAChRs play a crucial role in modulating the neurobiological pathways associated with addiction and smoking behavior. Patients with psychosis exhibit common genetic variance with tobacco addiction at the cholinergic receptor nicotinic alpha gene cluster, which encodes for nAChRs (60,61), and also less receptor expression (62), which could influence their higher smoking rates. These receptor differences may not translate into detectable structural changes in brain morphology by MRI but could still have significant functional implications, underscoring the importance of considering neurochemical receptor profiles in future research on smoking behavior and psychosis.

We observed a significant decrease in SFG, ACC, and insula gray matter volumes with increasing age regardless of group status. This could reflect normal developmental changes. The cortex is known to undergo synaptic pruning and maturation processes during late adolescence and early adulthood, when excess neurons and synaptic connections are eliminated, which results in a reduction in gray matter volume (63).

The main strength of the current study is the inclusion of a large cohort of CHR-P individuals and the unique opportunity to look at longitudinal data, which increases the power to detect possible changes. Despite dropouts over the course of the study, we were able to include a relatively high number of participants in analyses. Furthermore, the incorporation of data from 9 different sites enhances the generalizability of our findings. However, in addition to the limitations mentioned above, the current study has several other limitations that should be acknowledged. Our study did not account for medication use, which could affect the gray matter volume (64). However, few CHR-P participants used antipsychotic medication, and when they did, the doses were low, with no significant differences in medication use or dosage between CHR-P smokers and nonsmokers (Table 1). Furthermore, given the relatively young age of our sample, it is unlikely that medication use was prolonged, and thus it is unlikely that medication use had a substantial impact on the current findings. Third, the low conversion rate to psychosis (11%) might have precluded an effect of smoking in the converters, as structural brain differences are generally more prominent in individuals who convert than in nonconverters (18,26). Fourth, the absence of smoking control participants limited our ability to examine whether smoking-related brain volume changes differed between CHR-P individuals and control participants. While most research on smoking and brain volume has focused on older populations and revealed widespread gray matter volume reductions, a recent study of young adults reported reduced gray matter volume in smokers in regions such as the fusiform gyrus, inferior temporal gyrus, middle temporal gyrus, middle cingulate gyrus, and ACC compared with control participants (65). Future studies with matched control participants may clarify whether smoking-related brain volume reductions observed in control participants also apply to CHR-P individuals or whether the lack of association is specific to this group. Lastly, without data regarding the onset of smoking, we were unable to calculate pack years, an important measure that combines both smoking intensity and duration. This metric could provide a more sensitive assessment of smoking behavior, potentially revealing nuanced differences.

### Conclusions

The current findings do not support the notion that relatively mild smoking is associated with baseline or 8-month follow-up gray matter differences in CHR-P individuals or indicate a dose-response relationship within this population. This suggests that no tobacco-related associations with gray matter volume reductions are evident yet in this vulnerable group. Future research should extend the current findings in a sample with higher smoking intensity and longer follow-up intervals and explore alternative processes (with fMRI, white matter imaging, or nAChR imaging) beyond structural changes.

## Supplementary Material

Supplemental Text, Figures S1–S4, and Tables S1–S12

Key Resource Table
