# Supplementary material for: Tobacco Smoking and Gray Matter Volume in Individuals at Clinical High Risk for Psychosis: A Longitudinal Magnetic Resonance Imaging Study
Source: Biol Psychiatry Glob Open Sci. 2025 May 27;5(5):100539. doi: 10.1016/j.bpsgos.2025.100539 (PMC12270062; doi:10.1016/j.bpsgos.2025.100539)
Supplement: Supplemental Text, Figures S1–S4, and Tables S1–S12 [file mmc1.pdf]

SUPPLEMENTARY INFORMATION

Tobacco Smoking and Gray Matter Volume in Individuals at Clinical High-Risk for Psychosis: A Longitudinal Magnetic Resonance Imaging Study

Koster *et al.*

Supplementary information S1 Deviations from pre-registered analysis plan

1. Our initial baseline VBM analysis revealed no significant differences, preventing us from identifying data-driven regions of interest (ROIs) for longitudinal analyses. As outlined in our pre-registered plan, in the absence of such findings, we selected ROIs based on prior evidence of their involvement in both CHR-P individuals and smoking, including the prefrontal cortex and the insula. Within the prefrontal cortex, we focused on the superior frontal gyrus and additionally included the anterior cingulate cortex. Thus, our final ROIs were the superior frontal gyrus, insula, and anterior cingulate cortex.
2. To address the effects of an unbalanced distribution of CHR-P smokers and nonsmokers, we conducted a sensitivity analysis using equally distributed samples matched for age, sex, and cannabis use. These additional analyses were included to enhance the robustness of our findings.
3. Originally we stated that we would examine longitudinal changes using a group and group*time effect. However as our sample consists of adolescents during which pronounced brain developmental changes going on, we used age as key predictor in our models instead of timepoint.
4. To determine the strength of the reported evidence, we calculated Bayes factors. This enabled us to assess whether the data provided support for the null hypothesis, rather than simply failing to reject it.
5. In addition to the pre-registered longitudinal analysis, we performed linear mixed-effects models using past-month tobacco frequency as a fixed effect instead of smoking status. This additional analysis, which was not included in the original pre-registered plan, was added to explore potential dose-response relationships within the CHR-P group by substituting smoking status with past-month tobacco frequency.

Supplementary information S2 Bayes Factor calculation and interpretation

The bayesfactor_models() function of the bayestestR package (version 0.15.2) was used for Bayes Factor calculation, comparing the models with smoking behavior to the models without smoking behavior. The full models with smoking behavior assessed the main and interaction effects of smoking and age (baseline, 2-month, 4-month 6-month, 8-month follow-up) on SFG, ACC or insula grey matter volume. The null models without smoking behavior assessed the main and interaction effects of age on SFG, ACC or insula grey matter volume.

Thresholds for interpretation of Bayes Factors

Bayes Factor	Interpretation
1	No evidence for null hypothesis
1/3	Anecdotal evidence for null hypothesis
1/3 - 1/10	Moderate evidence for null hypothesis
1/10 - 1/30	Strong evidence for null hypothesis
1/30 - 1/100	Very strong evidence for null hypothesis
< 1/100	Extreme evidence for null hypothesis

Table S1. Number of baseline and follow-up measurements per group

	Baseline	2 month FU	4 month FU	6 month FU	8 month FU
Controls	81 (13%)	63 (17%)	63 (19%)	52 (18%)	52 (17%)
Non-smoking CHR-P	432 (69%)	245 (64%)	204 (61%)	183 (63%)	179 (60%)
Smoking CHR-P	110 (18%)	73 (19%)	66 (20%)	56 (19%)	69 (23%)
Total	623	381	333	291	300

Smokers were defined as those who smoke at least occasionally (≥ 1 on the tobacco severity scale), and non-smokers as participants who do not smoke at all (0 on the tobacco severity scale). Participants were classified as smokers from the moment they start smoking. Abbreviations: CHR-P, clinical high-risk for psychosis participants; FU, follow-up.

Table S2. CHR-P participants categorized per smoking behavior across the baseline and follow-up measurements.

	Baseline	2 months	4 months	6 months	8 months
**Tobacco addiction scale (n, %)**					
1	432 (80%)	245 (77%)	204 (76%)	183 (76%)	179 (72%)
2	96 (18%)	67 (21%)	63 (23%)	56 (23%)	66 (27%)
3	6 (1%)	2 (1%)	0 (0%)	0 (0%)	2 (1%)
4	8 (1%)	4 (1%)	3 (1%)	0 (0%)	1 (0%)
**Tobacco severity scale (n, %)**					
0	432 (80%)	245 (77%)	204 (76%)	183 (77%)	179 (72%)
1	64 (12%)	47 (15%)	42 (16%)	33 (14%)	36 (15%)
2	26 (5%)	16 (5%)	15 (6%)	15 (6%)	20 (8%)
3	18 (3%)	7 (2%)	8 (3%)	8 (3%)	11 (4%)
4	2 (0%)	3 (1%)	1 (0%)	0 (0%)	2 (1%)

Tobacco addiction scale, assessed by clinician: 1= abstinent, 2= use without impairment, 3= abuse, 4= dependence; Tobacco smoking severity scale, through self-report: 0=no use, 1= occasionally, 2= less than 10 cigarettes per day, 3= Between 10 and 25 cigarettes per day, 4= more than 25 cigarettes per day. Abbreviations: CHR-P, clinical high-risk for psychosis participants.

Table S3. SFG: Longitudinal analysis of smoking and age-related differences in SFG grey matter volume in CHR-P individuals

Insula	Estimate	SE	Df	t	P
Intercept	30.050	0.329	547	91.238	**<0.001**
Group	0.003	0.099	1352	0.029	0.977
Age	-0.029	0.002	246	-13.464	**<0.001**
Sex (Male)	-0.408	0.221	592	-1.847	0.065
Total intracranial volume	0.02	0.001	604	27.458	**<0.001**
Cannabis status (user)	-0.098	0.094	1389	-1.038	0.299
Site 2	0.797	0.425	469	1.877	0.061
Site 3	0.581	0.472	513	1.231	0.219
Site 4	-0.122	0.422	530	-0.289	0.773
Site 5	0.672	0.391	498	1.72	0.086
Site 6	0.549	0.421	513	1.305	0.192
Site 7	1.417	0.399	532	3.546	**<0.001**
Site 8	0.738	0.415	517	1.776	0.076
Site 9	-0.357	0.396	531	-0.900	0.369
Group x Age	0.002	0.0022	970	0.870	0.384

Fixed effects in the models were smoking status*age, sex, total intracranial volume, cannabis use and MRI site. Random effects were intercepts for subjects and random slopes for age at the subject level. Significant p-values are in bold. Abbreviations: CHR-P, clinical high-risk for psychosis; df, degrees of freedom; SE, standard error.

Table S4. SFG: Longitudinal analysis of smoking frequency and age-related differences in SFG grey matter volume in CHR-P individuals

**SFG**	**Estimate**	**SE**	**Df**	**t**	**P**
Intercept	30.05	0.329	547	91.276	**<0.001**
Low frequency	0.056	0.104	1305	0.537	0.591
Moderate-heavy frequency	-0.160	0.146	1350	-1.095	0.274
Age	-0.029	0.002	246	-13.437	**<0.001**
Sex (Male)	-0.405	0.221	593	-1.83	0.068
Total intracranial volume	0.020	0.001	604	27.455	**<0.001**
Cannabis status (user)	-0.092	0.094	1386	-0.98	0.327
Site 2	0.800	0.425	468	1.886	0.060
Site 3	0.589	0.472	513	1.248	0.213
Site 4	-0.121	0.422	530	-0.287	0.774
Site 5	0.673	0.390	498	1.724	0.085
Site 6	0.545	0.421	513	1.296	0.195
Site 7	1.435	0.400	533	3.591	**<0.001**
Site 8	0.743	0.415	517	1.79	0.074
Site 9	-0.352	0.397	532	-0.887	0.375
Low frequency x Age	0.001	0.002	1078	0.335	0.737
Moderate-heavy frequency x Age	0.005	0.003	954	1.606	0.109

Fixed effects in the models were group*age, sex, total intracranial volume, cannabis use and MRI site. Random effects were intercepts for subjects and random slopes for age at the subject level. Significant p-values are in bold. Abbreviations: CHR-P, clinical high-risk for psychosis; df, degrees of freedom; SE, standard error.

Table S5. ACC: Longitudinal analysis of smoking and age-related differences in ACC grey matter volume in CHR-P individuals

**ACC**	**Estimate**	**SE**	**Df**	**t**	**P**
Intercept	12.180	0.158	545	76.982	**<0.001**
Group	0.055	0.044	1322	1.24	0.215
Age	-0.009	0.001	235	-8.623	**<0.001**
Sex (Male)	-0.270	0.106	598	-2.552	**<0.001**
Total intracranial volume	0.008	0.000	618	22.953	**<0.001**
Cannabis status (users)	-0.005	0.042	1357	-0.128	0.898
Site 2	-0.049	0.202	448	-0.243	0.808
Site 3	0.155	0.227	505	0.686	0.493
Site 4	-0.192	0.204	531	-0.945	0.345
Site 5	0.1707	0.187	490	0.912	0.362
Site 6	-0.131	0.202	508	-0.648	0.518
Site 7	0.295	0.193	535	1.534	0.126
Site 8	0.283	0.200	524	1.416	0.157
Site 9	0.187	0.191	530	0.978	0.328
Group x Age	0.001	0.001	937	0.758	0.449

Fixed effects in the models were smoking status*age, sex, total intracranial volume, cannabis use and MRI site. Random effects were intercepts for subjects and random slopes for age at the subject level. Significant p-values are in bold. Abbreviations: ACC, anterior cingulate cortex; CHR-P, clinical high-risk for psychosis; df, degrees of freedom; SE, standard error.

Table S6. ACC: Longitudinal analysis of smoking frequency and age-related differences in ACC grey matter volume in CHR-P individuals

ACC	Estimate	SE	Df	t	P
Intercept	12.180	0.158	545	76.924	**<0.001**
Low frequency	0.075	0.047	1278	1.596	0.111
Moderate-heavy frequency	-0.011	0.066	1322	-0.164	0.870
Age	-0.008	0.001	235	-8.529	**<0.001**
Sex (Male)	-0.266	0.106	599	-2.508	**0.012**
Total intracranial volume	0.008	0.000	618	22.925	**<0.001**
Cannabis status (user)	-0.003	0.042	1353	-0.064	0.949
Site 2	-0.049	0.202	448	-0.242	0.809
Site 3	0.156	0.227	505	0.687	0.492
Site 4	-0.195	0.204	531	-0.957	0.339
Site 5	0.170	0.188	490	0.909	0.364
Site 6	-0.134	0.202	508	-0.664	0.507
Site 7	0.303	0.193	536	1.574	0.116
Site 8	0.285	0.201	523	1.422	0.156
Site 9	0.184	0.191	530	0.966	0.335
Low frequency x Age	0.001	0.001	1051	0.605	0.545
Moderate-heavy frequency x Age	0.001	0.001	922	0.792	0.429

Fixed effects in the models were group*age, sex, total intracranial volume, cannabis use and MRI site. Random effects were intercepts for subjects and random slopes for age at the subject level. Significant p-values are in bold. Abbreviations: ACC, anterior cingulate cortex; CHR-P, clinical high-risk for psychosis; df, degrees of freedom; SE, standard error.

Table S7. Insula: Longitudinal analysis of smoking and age-related differences in insula grey matter volume in CHR-P individuals

Insula	Estimate	SE	Df	t	P
Intercept	16.890	0.167	537	101.134	**<0.001**
Group	-0.013	0.054	1392	-0.236	0.814
Age	-0.011	0.001	255	-10.041	**<0.001**
Sex (Male)	-0.051	0.112	561	-0.458	0.647
Total intracranial volume	0.009	0.000	570	24.879	**<0.001**
Cannabis status (users)	0.041	0.051	1431	0.799	0.424
Site 2	0.149	0.213	443	0.703	0.482
Site 3	0.055	0.237	487	0.234	0.815
Site 4	-0.205	0.215	527	-0.954	0.341
Site 5	0.612	0.195	467	3.14	0.002
Site 6	-0.559	0.211	484	-2.65	0.008
Site 7	-0.277	0.204	538	-1.358	0.175
Site 8	0.401	0.214	541	1.877	0.061
Site 9	0.748	0.201	522	3.724	**<0.001**
Group x Age	0.002	0.001	940	1.504	0.133

Fixed effects in the models were smoking status*age, sex, total intracranial volume, cannabis use and MRI site. Random effects were intercepts for subjects and random slopes for age at the subject level. Significant p-values are in bold. Abbreviations: CHR-P, clinical high-risk for psychosis; df, degrees of freedom; SE, standard error.

Table S8. Insula: Longitudinal analysis of smoking frequency and age-related differences in insula grey matter volume in CHR-P individuals

**Insula**	**Estimate**	**SE**	**Df**	**t**	**P**
Intercept	16.880	0.167	537	101.177	**<0.001**
Low frequency	0.031	0.057	1341	0.539	0.590
Moderate-heavy frequency	-0.147	0.079	1385	-1.854	0.064
Age	-0.011	0.001	253	-9.989	**<0.001**
Sex (Male)	-0.045	0.112	564	-0.401	0.689
Total intracranial volume	0.009	0.000	570	24.87	**<0.001**
Cannabis status (user)	0.046	0.051	1428	0.902	0.367
Site 2	0.152	0.213	443	0.715	0.475
Site 3	0.057	0.237	487	0.243	0.808
Site 4	-0.208	0.215	528	-0.967	0.334
Site 5	0.612	0.195	468	3.141	0.002
Site 6	-0.565	0.211	485	-2.679	0.008
Site 7	-0.263	0.204	539	-1.289	0.198
Site 8	0.405	0.214	540	1.894	0.059
Site 9	0.748	0.201	523	3.726	**<0.001**
Low frequency x Age	0.001	0.001	1068	0.938	0.348
Moderate-heavy frequency x Age	0.003	0.002	937	2.097	**0.036**

Fixed effects in the models were group*age, sex, total intracranial volume, cannabis use and MRI site. Random effects were intercepts for subjects and random slopes for age at the subject level. Significant p-values are in bold. Abbreviations: CHR-P, clinical high-risk for psychosis; df, degrees of freedom; SE, standard error.

Supplementary information regarding CHR-P versus controls

The NAPLS dataset also contains controls data. However, since only 6 controls smoked, be opted to exclude smoking controls from the analysis. Thus, we carried out pre-registered exploratory analyses of baseline gray matter volume, cortical thickness, and gyrification index differences between CHR-P and controls, but moved these to the supplement as they do not directly address smoking-related questions.

Methods

In addition to exclusion criteria for CHR-P participants (current or lifetime DSM-5 Axis I psychotic disorder diagnosis, including affective psychoses; intelligent quotient <70; history of a central nervous system disorder; or psychosis-risk symptoms that are attributable to an Axis I disorder), controls were excluded if they: 1) met criteria for any psychosis risk-syndrome; current or past psychotic disorder, or Cluster A personality disorder; 2) had a family history (in first-degree relatives) of any psychotic disorder or disorder involving psychotic symptoms; or 3) were currently using psychotropic medication. See figure S1 for an exclusion flowchart and further reasons for exclusion. Analyses were performed as described in the main paper.

Results

81 non-smoking controls were included (Table S9). See Figure S1 for an exclusion flowchart and further reasons for exclusion. Since only six controls smoked, we opted to exclude smoking controls from the analyses. Thus, smoking controls at baseline (n=6), and if they started smoking at follow-up (n=2 at M2, n=1 at M4, n=1 at M8), were excluded. Scans of participating controls performed before the start of smoking were retained.

CHR-P participants showed larger gray matter volume compared to controls widespread throughout the brain at baseline (Figure S4; Table S12). In CHR-P participants, cortical thickness was significantly larger than controls across the brain (Table S10, Figure S2), with no regions showing increased or decreased thickness in controls. Furter, gyrification was significantly larger in controls compared to CHR-P participants across the brain (Table S11, Figure S3), with no regions showing increased or decreased gyrification in CHR-P participants.

Supplementary discussion regarding CHR vs controls

In our exploratory analyses we observed larger grey matter volume and cortical thickness at baseline in CHR-P participants compared to controls. These findings are unexpected, as existing CHR-P literature typically reports decreases in these measures compared to controls (1-3). However, our CHR-P participants were relatively younger than those in these previous studies, which may suggest that they have not experienced the same degree of neurodevelopmental changes (i.e. delayed synaptic pruning) or damage. However, Collins et al. (2023) also demonstrated larger cortical thickness at baseline in CHR-P participants compared to controls within the same dataset. Authors demonstrated that CHR-P participants who later converted to psychosis exhibited accelerated cortical thinning compared to both non-converters and controls (4). Gyrification was significantly larger in controls compared to CHR-P participants in 2 clusters covering the SFG and caudal ACC, and the precentral gyrus. Smaller gyrification index in CHR-P individuals indicates decreased cortical folding, which may be associated with less cortical complexity and efficiency in neural connectivity (5). Reduced but also increased brain gyrification has been shown in schizophrenia and high-risk individuals (6). As changes in cortical gyrification may indicate disrupted neural connectivity during brain development(7), the reduced gyrification observed in CHR-P participants may indicate disruptions in early neurodevelopmental processes, potentially contributing to the pathophysiology. It is important to note that these results were derived from exploratory analyses, and further research is necessary to evaluate or explain these findings.

Figure S1. Number of participants and reasons for exclusion for the analysis. Scans of participants without baseline MRI were excluded (n=2 at M2, n=3 at M4, n=3 at M6, n=4 at M8). Further, smoking controls were excluded at baseline (n=6), and if they started smoking at follow-up (n=2 at M2, n=1 at M4, n=1 at M8), that scan and all subsequent scans were removed, but prior scans were retained. Additionally, 3 participants were excluded due to preprocessing errors (n=1 control at M2, n=1 CHR-P at M4, n=2 controls at M8). Abbreviations: FU, follow-up.

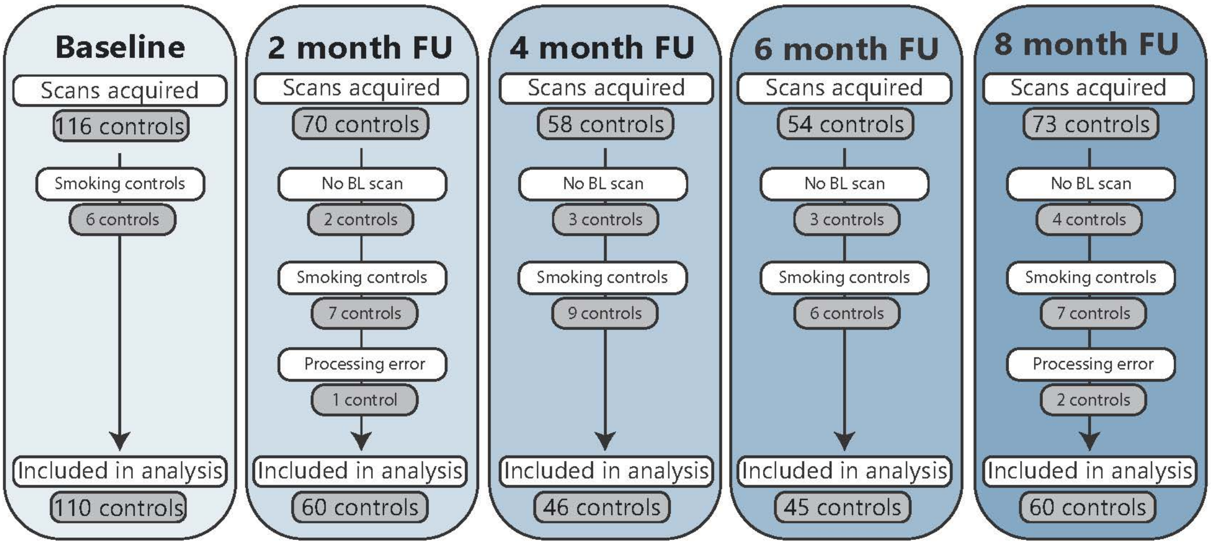

Table S9 Demographical and clinical characteristics of CHR-P participants and controls at baseline.

	**CHR-P (n=542)**	**Controls (n=81)**	**p**
Age in years[a]	18.4 ± 3.97	18.5 ± 4.22	0.939b
Sex (N female, %)	235 (43%)	41 (51%)	0.268[c]
Years of education[a]	11.7 ± 3.03[d]	12.1 ± 3.48	0.181[b]
Smoking frequency (N, %)			**<0.001**[c]
No use	432 (80%)	81 (100%)	
Occasionally	64 (12%)	0 (0%)	
<10 cigarettes per day	26 (5%)	0 (0%)	
10-25 cigarettes per day	18 (3%)	0 (0%)	
>25 cigarettes per day	2 (0%)	0 (0%)	
Cannabis users (N, %)	143 (26%)	7 (9%)	**<0.001**[c]
Cannabis use severity (N, %)			**0.023**[c]
No use	399 (74%)	74 (91%)	
Once/twice per month	62 (11%)	4 (5%)	
3-4 times per month	18 (3%)	0 (0%)	
1-2 times per week	26 (5%)	1 (1%)	
3-4 times per week	22 (4%)	2 (2%)	
Almost daily	15 (3%)	0 (0%)	
Alcohol users (N, %)	208 (38%)	36 (44%)	0.357[b]
Alcohol use severity (N, %)			0.423[b]
No use	334 (62%)	45 (56%)	
Once/twice per month	85 (16%)	17 (21%)	
3-4 times per month	49 (9%)	8 (10%)	
1-2 times per week	40 (7%)	9 (11%)	
3-4 times per week	27 (5%)	2 (2%)	
Almost daily	7 (1%)	0 (0%)	
Psychotic symptom severity[a]			
Total negative symptoms	12.2 ± 6.36[e]	1.44 ± 2.16	**<0.001**[b]
Total positive symptoms	12.8 ± 3.37[d]	0.815 ± 1.21	**<0.001**[b]
Total general symptoms	9.33 ± 4.22[f]	1.23 ± 2.08	**<0.001**[b]
Total disorganization symptoms	5.14 ± 3.21[e]	0.568 ± 0.987	**<0.001**[b]
Antipsychotic users (N, %)	113 (21%)	0 (0%)	**<0.001**[c]
Antipsychotic medication dosage[a] (mg/day CPZ)	160 ± 174	0	**0.004**[b]

All controls were non-smokers. Abbreviations: CHR-P, clinical high-risk for psychosis participants; CPZ, chlorpromazine. Significant p-values are in bold.

[a] Values are mean ± standard deviation

[b] Independent t-test.

[c] Chi-squared test

[d] Data were missing for 1 person

[e] Data were missing for 10 people

[f] Data were missing for 11 people

Figure S2. Cortical thickness in the CHR-P participants compared to controls.
Figure shows localization of significant greater cortical thickness ($p < 0.01$, TFCE, FWE-corrected) CHR-P participants compared to controls. Greater cortical thickness comprises frontal, parietal, temporal, and occipital regions.

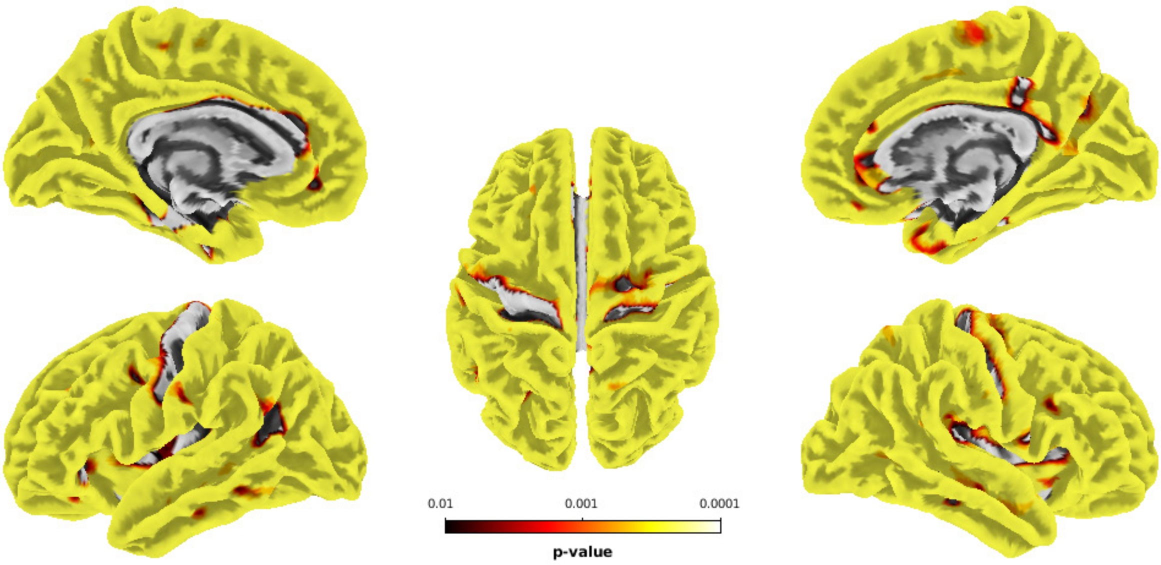

Table S10. Brain regions showing greater cortical thickness in CHR-P participants versus controls.

Hemisphere	P	Size (vertices)	Overlap	Region
Left	<0.001	28437	8%	Superior frontal
			7%	Superior parietal
			7%	Inferior parietal
			7%	Postcentral
			6%	Supramarginal
			6%	Precentral
			5%	Rostral middle frontal
			5%	Precuneus
			5%	Superior temporal
			4%	Lateral occipital
			4%	Middle temporal
			3%	Fusiform
			3%	Inferior temporal
			3%	Lingual
			3%	Caudal middle frontal
			2%	Lateral orbitofrontal
			2%	insula
			2%	Posterior cingulate
			2%	Pars opercularis
			2%	Medial orbitofrontal
			2%	Pars triangularis
			1%	Parahippocampal
			1%	bankssts
			1%	Perical carine
			1%	Cuneus
			1%	Cauldal anterior cingulate
Right	<0.001	28310	8%	Superior frontal
			7%	Superior parietal
			7%	Inferior parietal

7%	Postcentral
6%	Supramarginal
6%	Precentral
5%	Rostral middle frontal
5%	Precuneus
5%	Superior temporal
4%	Lateral occipital
4%	Middle temporal
3%	Fusiform
3%	Inferior temporal
3%	Lingual
3%	Caudal middle frontal
2%	Lateral orbitofrontal
2%	insula
2%	Posterior cingulate
2%	Pars opercularis
2%	Medial orbitofrontal
2%	Pars triangularis
1%	Parahippocampal
1%	bankssts
1%	Perical carine
1%	Cuneus
1%	Cauldal anterior cingulate

P-values are FWE-corrected values after applying threshold-free cluster enhancement (TFCE, $p < 0.01$). Covariates were sex, age, age^2, total intracranial volume, cannabis use and MRI site. Region-column shows the label according to the CAT12 output.

Figure S3. Gyrification index in controls compared to CHR-P participants

Figure shows localization of significant greater gyrification index ($p < 0.01$, TFCE, FWE-corrected) in controls compared to CHR-P participants

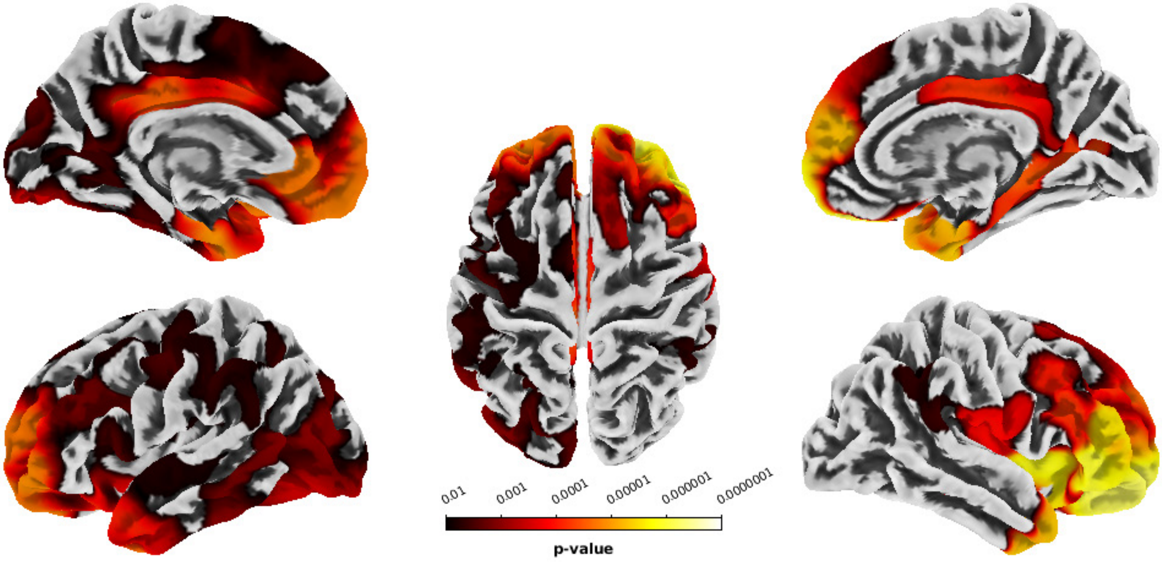

Table S11. Brain regions showing greater gyrification index in controls compared to CHR-P participants.

Hemisphere	P	Cluster-size	Overlap	Region
Left	<0.001	15379	9[illegible]%	Rostral middle frontal
			9%	Superior frontal
			7%	Lateral occipital
			6%	Supra marginal
			5%	Precentral gyrus
			5%	Lateral orbitofrontal
			4%	Superior temporal
			4%	Fusiform
			4%	Lingual
			4%	Inferior temporal
			4%	Superior parietal
			4%	Posterior cingulate
			3%	Middle temporal
			3%	Pars opercularis
			3%	Inferior parietal
			3%	Insula
			3%	Isthmus cingulate
			3%	Post central
			3%	Precuneus
			2%	Medial corbitofrontal
			2%	Caudal middle frontal
			2%	Rostral anterior cingulate
			1%	Caudal middle frontal
			1%	Entorhinal
			1%	Paracentral
Right	<0.001	10085	15%	Rostral middle frontal
			10%	Superior frontal
			8%	Insula
			7%	Supramarginal
			7%	Lateral orbitofrontal
			5%	Posterior cingulate
			5%	Isthmus cingulate
			4%	Pars triangularis
			4%	Post central
			4%	Parahippocampal
			4%	Superior temporal
			3%	Caudal middle frontal
			2%	Precuneus
			2%	Medial orbitofrontal
			2%	Fusiform
			2%	Pars orbitalis
			2%	Inferior parietal
			2%	Lingual
			2%	Pars opervularis
			2%	Precentral
			2%	Pericalcarine
			2%	Temporal pole
			2%	Enorhinal
			1%	Inferior temporal

P-values are FWE-corrected values after applying threshold-free cluster enhancement (TFCE, $p < 0.01$). Covariates were sex, age, age^2, total intracranial volume, cannabis use and MRI site. Region-column shows the label according to the CAT12 output.

Figure S4. Areas of significantly increased grey matter in the CHR-P group compared to the control group. Results show the FWE-corrected p-value map ($p < 0.01$, TFCE). The colour bar on the right represents T-values with increased response (red/yellow). Results are overlaid on a standardized T1-weighted image. Only clusters with a cluster size of 10 or more voxels are shown.

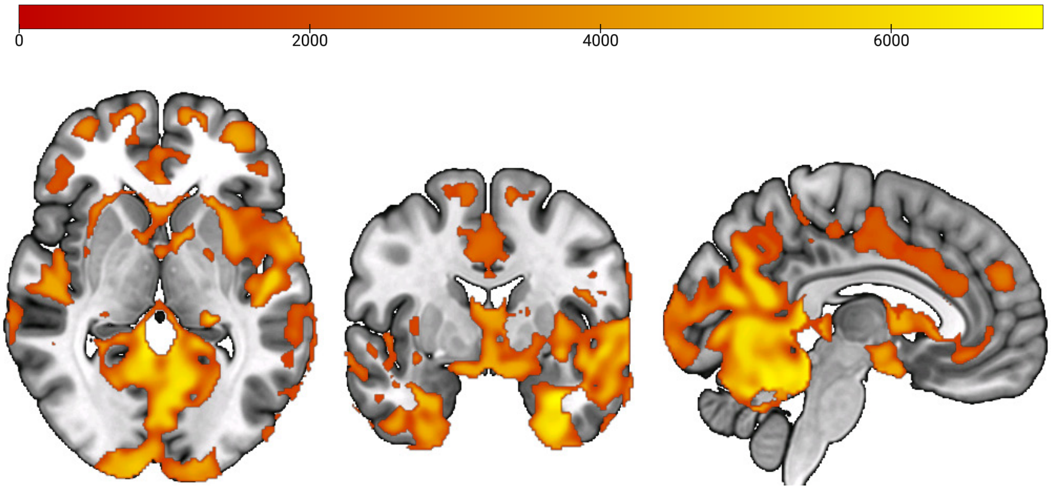

Table S12. Significant clusters resulting from voxel-based morphometry analysis comparing CHR-P participants and controls.

Region	Voxels	MNI coordinates			TFCE	Peak p-value
		x	y	z		
**CHR-P > Controls**						
Widespread	132472	-3	-39	-12	6860	<0.001
L inferior temporal gyrus	26	-58	-58	8	1696	0.009

Results are listed for p<0.01 FWE and a cluster size of 10 or more voxels. Covariates were sex, age, age^2, total intracranial volume, cannabis use and MRI site. Regions were labelled according to the maximum probability tissue atlas in CAT12 with labelled data provided by Neuromorphometrics (http://neuromorphometrics.com/). Abbreviations: CHR-P, clinical high-risk for psychosis participants; FWE, family-wise error; L, left; MNI, Montreal Neurological Institute; R, right; TFCE, threshold-free cluster enhancement.

References

1. Group ECHRfPW (2021): Association of Structural Magnetic Resonance Imaging Measures With Psychosis Onset in Individuals at Clinical High Risk for Developing Psychosis: An ENIGMA Working Group Mega-analysis. *JAMA Psychiatry*. 78:753–766.
2. Merritt K, Luque Laguna P, Irfan A, David AS (2021): Longitudinal Structural MRI Findings in Individuals at Genetic and Clinical High Risk for Psychosis: A Systematic Review. *Frontiers in Psychiatry*. 12:620401.
3. Luna LP, Radua J, Fortea L, Sugranyes G, Fortea A, Fusar-Poli P, et al. (2022): A systematic review and meta-analysis of structural and functional brain alterations in individuals with genetic and clinical high-risk for psychosis and bipolar disorder. *Progress in neuro-psychopharmacology & biological psychiatry*. 117:110540.
4. Collins MA, Ji JL, Chung Y, Lympus CA, Afriyie-Agyemang Y, Addington JM, et al. (2023): Accelerated cortical thinning precedes and predicts conversion to psychosis: The NAPLS3 longitudinal study of youth at clinical high-risk. *Molecular psychiatry*. 28:1182–1189.
5. White T, Su S, schmidt M, Kao CY, Sapiro G (2010): The development of gyrification in childhood and adolescence. *Brain and cognition*. 72:36–45.
6. Matsuda Y, Ohi K (2018): Cortical gyrification in schizophrenia: current perspectives. *Neuropsychiatric disease and treatment*. 14:1861–1869.
7. Razavi MJ, Zhang T, Liu T, Wang X (2015): Cortical Folding Pattern and its Consistency Induced by Biological Growth. *Scientific reports*. 5:14477.
